# Alone

**DOI:** 10.1017/S1478951526102351

**Published:** 2026-04-20

**Authors:** Miguel Julião

**Affiliations:** Equipa Comunitária de Suporte em Cuidados Paliativos PalCo, ULS Amadora/Sintra, Portugal

When she enters,
you stand alone.
Visceral encounter.
You facing you.
Alone.

No hands of comfort
reaching out for you.
No voice breaking the anguish
in your chest.

There will be more cigarettes in a pack,
than friends visiting you.
Smoke curls where words should be,
endless conversations,
– not human –, you’ll see.

It’s a cachexia of the soul,
a thinning no one sees.

And still, you remain,
unmoving,
witness and wound.
You facing you.

